# MetAMDB: Metabolic Atom Mapping Database

**DOI:** 10.3390/metabo12020122

**Published:** 2022-01-27

**Authors:** Collin Starke, Andre Wegner

**Affiliations:** Department of Bioinformatics and Biochemistry, Braunschweig Integrated Centre of Systems Biology (BRICS), Technische Universität Braunschweig, Rebenring 56, 38106 Braunschweig, Germany; c.starke@tu-bs.de

**Keywords:** atom mappings, atom transitions, atom mapping model, systems biochemistry, ^13^C metabolic flux analysis, metabolic model, database, AAM, RXN

## Abstract

MetAMDB is an open-source metabolic atom mapping database, providing atom mappings for around 43,000 metabolic reactions. Each atom mapping can be inspected and downloaded either as an RXN file or as a graphic in SVG format. In addition, MetAMDB offers the possibility of automatically creating atom mapping models based on user-specified metabolic networks. These models can be of any size (small to genome-scale) and can subsequently be used in standard ${}^{13}$C metabolic flux analysis software.

## 1. Introduction

Metabolic fluxes are a valuable read-out for biomedical research [1] as well as for biotechnological applications [2]. However, metabolic fluxes are not directly measurable and must be inferred through metabolic modeling. Besides constraint-based modeling [3], the most successful approaches are based on stable isotope labeling experiments [4,5]. Here, a stable isotope (mostly ${}^{13}$C) labeled substrate is applied and the metabolization of this compound will lead to specific isotopic enrichment patterns in downstream metabolites. For example, ${}^{13}$C metabolic flux analysis combines experimentally determined isotopic enrichment patterns of metabolites with a metabolic atom mapping model to infer metabolic fluxes [6]. An atom mapping describes the one-to-one correspondence between a substrate atom and a product atom in a metabolic reaction [7]. Thus, within an atom mapping model, one can follow every atom throughout the metabolic network. The creation of such a model is a time-consuming process and requires detailed knowledge about the reaction mechanisms. Therefore, it is mostly limited to smaller simplified models of metabolism. There are a few resources available that provide computationally derived atom mappings for single reactions [8,9,10], but there is currently no resource available that can automatically generate custom atom mapping models.

Here, we present MetAMDB (Metabolic Atom Mapping Database), a freely accessible, web-resource for metabolic atom mappings (https://metamdb.tu-bs.de/ (accessed on 30 December 2021)). Atom mappings for individual reactions can be inspected and downloaded either as an RXN file or as a graphic in SVG format. Besides computationally derived atom mappings, MetAMDB provides a curated set of reactions (around 1000) that cover central carbon metabolism. MetAMDB complements the existing atom mapping resources such as MetaCyc, as it provides around 27,000 atom mappings that are not present in any other current database. In addition, users can submit custom metabolic models and download the corresponding carbon atom mapping models. As such, MetAMDB will greatly facilitate the use of bigger metabolic atom mapping models, as they now can be generated easily, even by non-experts. The MetAMDB source code is available at https://github.com/metamdb/metamdb (accessed on 30 December 2021) under the MIT licence.

## 2. Data Collection and Processing

**Figure 1:**
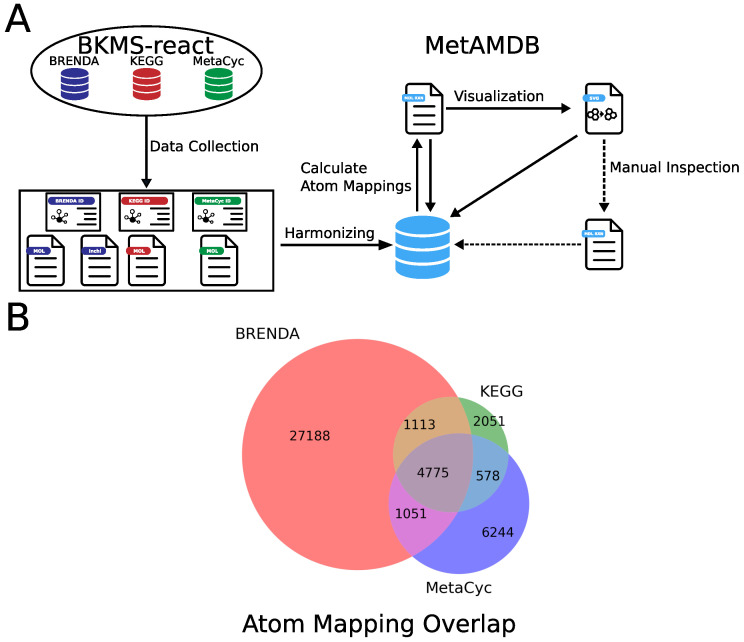
(**A**) All data were collected from BKMS-react. To avoid duplicates, metabolites were matched internally using a synonym list from BRENDA, and reactions were linked to a single reaction in MetAMDB based on BKMS-react reaction groups. With all reaction data available, atom mappings for each reaction were calculated and visualized. Afterward, a set of core reactions (around 1000) that cover central carbon metabolism were curated. (**B**) Venn diagram of all atom mappings in the MetAMDB database and their respective source database annotations.

The general overview of how we collected, processed, and curated the data included in MetAMDB is depicted in Figure 1A.

### 2.1. Collection of Reaction and Metabolite Data

To include a comprehensive set of reactions, we parsed reaction data from BKMS-react [11], a biochemical reaction database containing data mainly collected from the three databases BRENDA [12], KEGG [10], and MetaCyc [9]. BKMS-react integrated reaction data from these databases by comparing compound structures and names, thereby grouping reactions with the same substrates and products. Based on these data, we linked these groups to a single reaction in MetAMDB. In total, we included 75,000 reactions. To avoid duplicates, we matched metabolites internally using an updated synonym list from BRENDA and combined metabolites with different protonation states as this does not affect atom mappings. With this approach, we linked multiple reaction and metabolite identifiers of external databases to a single entry in the MetAMDB database. Multiple efforts have been made to harmonize compounds and reactions in metabolic databases [13,14]. Recently, Jin and colleagues developed a method based on a subgraph isomorphism algorithm and applied this method to harmonize reactions between KEGG and MetaCyc [14]. To further remove potential duplicate reactions, we compared the harmonized reactions obtained by Jin et al. to the reactions present in MetAMDB. Of the 2932 harmonized MetaCyc reactions, 495 had a missing link to a KEGG reaction in MetAMDB, which we subsequently added. This harmonization is advantageous to simplify the creation of atom mapping models because a mixture of identifiers from different databases can be used. We have decided to consider reactions in isolation from organisms because we assume that an enzyme with the same substrates and products, that is present in multiple organisms, will have identical atom mappings. Finally, we parsed metabolite structures in the form of MOL files from BRENDA. If BRENDA MOL files were not available, we considered MetaCyc or KEGG, depending on their availability.

### 2.2. Automated Generation of Atom Mappings

Atom mappings track every substrate atom in a reaction to their corresponding product atom. The difficulty of generating an atom mapping is therefore dependent on the complexity of the corresponding reaction. Many different atom mapping algorithms have been developed, like DREAM [15], CLCA [16], Pathway Tools Software [17], Reaction Decoder Tool (RDT) [18], and a recently developed automatic mapper [19], with respective advantages and disadvantages for particular enzyme classes [7]. We decided to use RDT for the automatic generation of atom mappings because RDT reached the overall highest accuracy in the study by Gonzalez et al. [7]. In addition, RDT is open-source, which makes it easy to modify and integrate into existing pipelines. RDT generates atom mappings in the form of RXN files from unmapped MOL files.

Using RDT, we were able to successfully calculate atom mappings for about 50,000 reactions. A problem we encountered was that RDT reordered atoms of the same metabolite randomly and inconsistently across multiple reactions, leading to confusing atom mappings. For this reason, we reordered all atoms in a molecule according to their InChI numbering [20]. This ensures that the atom sorting across all metabolites is consistent and reproducible. The remaining 25,000 reactions were either incomplete (missing substrates or products), contained an incorrect MOL file, or RDT did not converge within one hour. As a consequence, no atom mappings were calculated. As a final quality measure, we checked the carbon balance of all reactions, meaning the sum of all carbons of the substrates must be equal to the sum of carbons of all products. In total, we removed further 6500 reactions due to carbon unbalances, leaving around 43,000 reactions with atom mappings in MetAMDB.

MetaCyc and KEGG also provide computationally derived atom mappings. MetaCyc atom mappings, like MetAMDB atom mappings, are based on atom numberings of their respective compounds, while KEGG atom mappings use a custom format [21]. Because MetaCyc and KEGG already provide atom mappings for a number of reactions, we assessed the overlap with MetAMDB. MetAMDB provides atom mappings for around 27,000 reactions that are only present in BRENDA and not in MetaCyc or KEGG (Figure 1B), showing that MetAMDB is a valuable resource for atom mappings.

### 2.3. Automatic Generation of Atom Mapping Models

**Figure 2:**
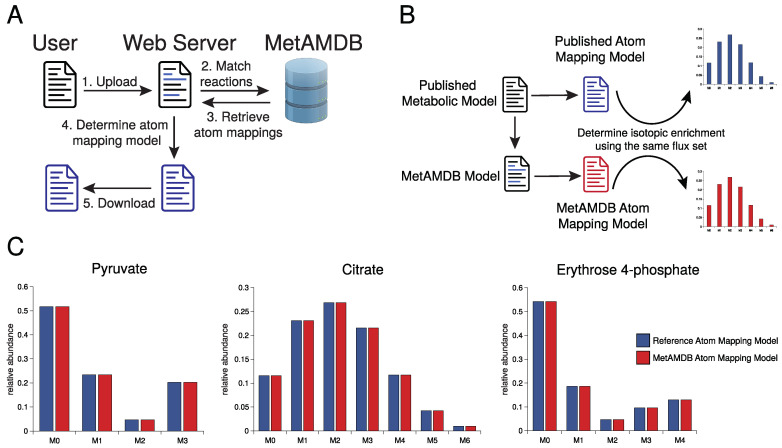
(**A**) Workflow for automated generation of atom mapping models. (**B**) MetAMDB atom mapping model test strategy. A published *E. coli* model [22] was compared with MetAMDB atom mappings for the same model and the same flux set using simulated isotopic enrichment patterns. (**C**) Simulation of isotopic enrichment for the metabolites pyruvate, citrate, and erythrose 4-phosphate using the published atom mapping model (blue) and MetAMDB derived atom mappings for the same metabolic network (red).

Metabolic atom mapping models are most widely used in ${}^{13}$C metabolic flux analysis. Since the creation of such a model requires detailed biochemical knowledge that goes beyond simple reaction stoichiometry, these models are usually limited to smaller simplified metabolic networks. Nevertheless, they provide the means to follow every atom throughout the metabolic network. This opens the possibility of applications beyond flux determination, in particular for genome-scale atom mapping models. Traditionally, these atom mapping models are specific for one element (e.g., carbon) and are described in the ABC format. The ABC format is a per element atom mapping representation based on case-sensitive letters. For example, the carbon atom mapping of the glutaminase reaction in ABC-format is ”glutamine (abcde) -> glutamate (abcde)”, meaning the glutamine carbon “a” maps to the glutamate carbon “a” and so forth. While this format is simple and easily human-readable, showing that the carbon backbone in the glutaminase reaction is not changing, it misses the mapping of the letter to the original atom in the molecule. Theoretically, the carbon “a” can be any of the 5 carbons of glutamine. That makes model generation even more error-prone for beginners. To overcome this problem, MetAMDB can automatically generate custom atom mapping models based on user-submitted metabolic models and database atom mappings (Figure 2A). Specifically, users upload a metabolic model, which is converted to a MetAMDB model. The MetAMDB algorithm then calculates the atom mapping model for the given metabolic model. The metabolic model format is a custom CSV-based format containing four columns: (1) reaction name, (2) substrates, (3) reversibility, (4) and products. Identifiers from either BRENDA, KEGG, or MetaCyc must be specified in square brackets for the reaction name, substrates, and products to match the reaction correctly to a MetAMDB reaction. The atom mapping models are calculated in the ABC-Format and displayed after each metabolite. More details can be found in the MetAMDB documentation (https://metamdb.github.io/docs/getting-started (accessed on 30 December 2021)).

To show that MetAMDB can automatically generate correct atom mapping models, we compared a published atom mapping model with a MetAMDB derived atom mapping model for the same metabolic network. To that end, we used the *E. coli* model of central carbon metabolism provided by INCA [22] (see Table S1). Initially, we considered comparing ABC-mappings provided by INCA directly to the ABC-mappings generated with MetAMDB. However, as described above, with the ABC format it is not directly clear to which atom in the original molecule the letter corresponds. Therefore, two different ABC-mappings can still be based on the same atom mapping. MetAMDB derived atom mappings are based on the InChI numbering, which the model provided by INCA, however, did not employ, making a direct comparison not possible. For that reason, we simulated the ${}^{13}$C isotopic enrichment for all metabolites of both models using the INCA software suite [23] and compared the simulated isotopic enrichment patterns (Figure 2B). These isotopic enrichment patterns are uniquely determined by the atom mapping model and the corresponding flux set. Using the same flux set for both models should therefore lead to identical enrichment patterns when the atom mappings are identical. First, we converted the INCA model into a MetAMDB readable model by adding MetaCyc identifiers (Table S2). To compare the atom mapping model from MetAMDB (Table S3) to the original model, we limited the number of carbons for acetyl-CoA to the acetyl moiety and removed symmetries as they were not present in the original model. Using the same flux set (Table S4) for both models, we obtained identical isotopic enrichment patterns for all metabolites included in the metabolic network (Figure 2C, Tables S5 and S6), showing that MetAMDB can automatically generate correct atom mapping models.

### 2.4. Why Curation of Atom Mappings Is Needed

**Figure 3:**
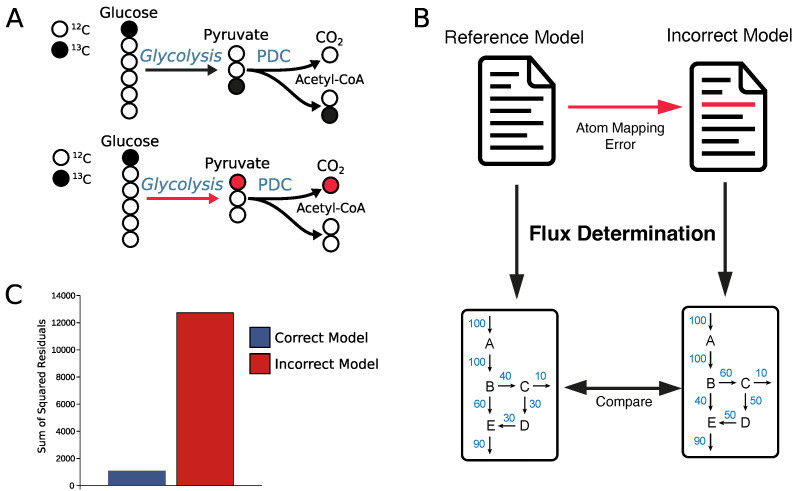
(**A**) Upper part: Glycolysis converts glucose into two molecules of pyruvate, which in turn is converted to acetyl-CoA and CO${}_{2}$. Usually, [1-${}^{13}$C${}_{1}$]glucose will label the third pyruvate carbon ([3-${}^{13}$C${}_{1}$]pyruvate). The first carbon is split off in the pyruvate dehydrogenase complex resulting in a labeled acetyl-CoA ([2-${}^{13}$C${}_{1}$]acetyl-CoA). Lower part: An atom mapping error in glycolysis was introduced, leading to [1-${}^{13}$C${}_{1}$] pyruvate instead of [3-${}^{13}$C${}_{1}$]pyruvate. This error then continues in the pyruvate dehydrogenase reaction, leading to labeling in carbon dioxide and not in acetyl-CoA. (**B**) Test workflow for the significance of an atom mapping error for flux determination. A single atom mapping error was introduced in the aldolase of the published *E. coli* model [22]. For both models a ${}^{13}$C metabolic flux analysis was performed and the influence on the determined fluxes was evaluated. (**C**) The sum-of-squared residuals (SSR) for the flux determination of the correct model (blue) and model with one error (red). The correct model SSR was about ten times (10x) lower than the SSR of the incorrect model.

RDT has a reported accuracy of around 90% (with some enzyme categories being better than others) [7], meaning approximately 10% of mappings contain an error. If one would look at a single reaction, this seems like an acceptable error rate, but it turns out to be rather problematic in a series of reactions such as a metabolic model. In a metabolic model, even one small error in a single reaction can lead to completely incorrect results. Suppose, we perform a stable isotope labeling experiment with [1-${}^{13}$C${}_{1}$]glucose as a tracer. With correct atom mappings, we can follow the labeled carbon atom to acetyl-CoA (Figure 3A). However, if there is an atom mapping error in just one of the 10 glycolytic reactions, the simulated labeling can drastically change. For example, an atom mapping error in the aldolase reaction (Figure S1) would move the ${}^{13}$C labeled atom away from acetyl-CoA to the carbon dioxide released by the pyruvate dehydrogenase complex (Figure 3A).

To test the impact of such an error, we again used the published *E. coli* model [22] from INCA and introduced a single error in glycolysis (see Table S7). We then performed a ${}^{13}$C metabolic flux analysis and determined the fluxes and compared the simulated isotopic enrichment patterns, sum-of-squared residuals, and flux distribution of the original model and the model containing the atom mapping error in glycolysis (Figure 3B, see Supplementary Information for method details).

Using a mixture of 75% [1-${}^{13}$C${}_{1}$] and 25% [U-${}^{13}$C${}_{6}$] labeled glucose as a tracer, the incorrect atom mapping model largely overestimated the M2 of pyruvate (Table S8) and subsequently the pyruvate dehydrogenase flux (Table S4). Overall, the estimated fluxes using the incorrect atom mapping model have much broader 95% confidence intervals (Table S4) and a much higher sum of squared residuals (Figure 3C), showing that the incorrect atom mapping model does not fit the data well. These results clearly demonstrate that one single error in the atom mapping model can lead to a completely different result, showing that error-free atom mapping models are fundamental for correct flux estimations. For this reason, we decided to curate a set of core reactions (around 1000) that cover central carbon metabolism. Of these, approximately 6% contained an error. It is also possible for MetAMDB users to curate atom mappings, which will be integrated into MetAMDB after review.

## 3. Database Content and Usage

MetAMDB provides atom mappings for reactions present in BKMS-react [11]. Every atom mapping is linked to their respective compounds and enzymes, and all of them are accessible by their KEGG, MetaCyc, or BRENDA identifiers. MetAMDB atom mappings are freely available on the public webpage (https://metamdb.tu-bs.de/ (accessed on 30 December 2021)). The database consists of approximately 43,000 reactions with atom mappings generated by RDT and corresponding structural images in SVG format. Around 1000 of these reactions were curated. Each atom mapping, if possible, is linked to a BRENDA, KEGG, and MetaCyc reaction.

To find a specific atom mapping, users can search MetAMDB using different database queries. For example, reaction identifiers (BRENDA, KEGG, MetaCyc) or reaction names can be used. Users can also search for specific substrates or products included in a reaction. We have implemented a fuzzy string search, meaning that glucose as a search term will also match reactions that include glucose-6-phosphate. A successful query will show the reaction name, the identifier, substrates and products, and whether the atom mapping was curated. Additional information is contained on the reaction page itself, which can be opened from the database query results. The atom mapping can be viewed (or downloaded) in the RXN file format. An image of the reaction structure, which includes the atom mapping can also be opened and downloaded. A help page with documentation for possible use cases can be found on the MetAMDB website.

**Figure 4:**
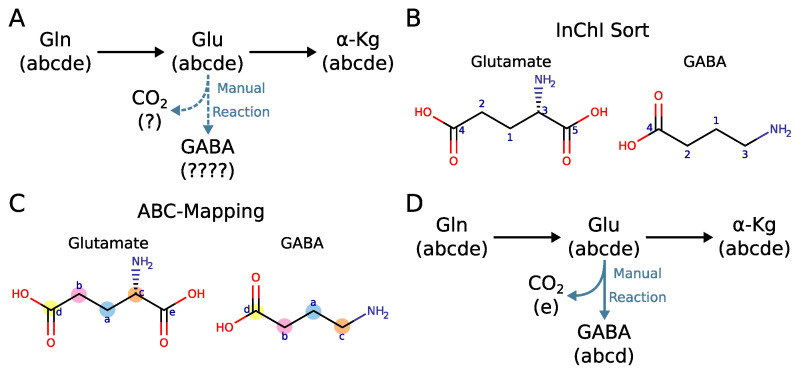
(**A**) Example of an existing ABC-format model where the reaction from glutamate to GABA is manually added. The atom mapping of the manual reaction is unknown since the sorting of the original glutamate is not known. (**B**) InChI numbering glutamate and GABA. (**C**) InChI numbering of glutamate and GABA converted to ABC-mappings. (**D**) ABC-format example with the manual reaction from glutamate to GABA if all metabolites are InChI sorted.

Users can upload custom metabolic models to obtain an atom mapping model for their specified reactions. If necessary, reactions can be simplified by omitting substrates or products from the reaction (for example, cofactors such as ADP and ATP). A successfully parsed metabolic model can be viewed on the reaction model page, which displays reactions and compounds with the subsequent linked MetAMDB pages. Atom mappings in the ABC format are also displayed, which if necessary can be manually adjusted. If a simplified reaction is needed (e.g. multiple linear reactions condensed into one), or a reaction that is not present in MetAMDB, reactions with custom identifiers can be written that require ABC atom mappings provided by the user. These atom mappings have to match the MetAMDB atom mappings of the rest of the model. Figure 4A depicts an example of how a potential reaction and the matching ABC mapping would be added to a MetAMDB model. Glutamine is converted to glutamate and subsequently to alpha-ketoglutarate without changing the carbon backbone. In case, a user wants to manually add the atom mapping for the reaction that converts glutamate to gamma-aminobutyric acid (GABA) to the existing model, one has to know to which carbon atoms of glutamate the “abcde” corresponds to. In MetAMDB the letters correspond to the InChI numbering (Figure 4B). With the InChI numbering both glutamate and GABA are sorted in the same way (Figure 4C). An established sorting algorithm ensures that each metabolite has a unique and reproducible atom numbering that can be understood and utilized by users for their own manual atom mappings (Figure 4D). The final model can be downloaded and used for further analysis and modeling applications.

Some of the functionality of MetAMDB can also be accessed through a REST-API. For example, reaction and pathway data are accessible, as well as the database search.

## 4. Conclusions

The quantitative analysis of metabolism is an important part of systems biology. These analyses are often based on stable isotope-labeled experiments and require an atom-resolved understanding of the metabolic network. That means one has to be able to follow the fate of every atom in the network. Creating such a model is a time-consuming and error-prone process, in particular for larger models. To overcome this problem, we developed MetAMDB. MetAMDB provides atom mappings for single reactions but also supports the automatic generation of atom mapping models. To our knowledge, this is the first implementation of such a feature. As such, MetAMDB will greatly facilitate the use of bigger (up to genome-scale) metabolic atom mapping models, as they can now be generated easily, even by non-experts. Since a single atom mapping error can make the entire downstream analysis faulty, we have curated a subset of reactions of central carbon metabolism and have integrated the possibility in MetAMDB that users can correct incorrect atom mappings.

## Data Availability

The metabolic models are available in the Supplementary Material. Calculated atom mappings are available on the MetAMDB website: https://metamdb.tu-bs.de (accessed on 30 December 2021). Source code of the MetAMDB software is available at GitHub: https://github.com/metamdb/metamdb (accessed on 30 December 2021). Documentation for the MetAMDB website and API can be found in the MetAMDB documentation: https://metamdb.github.io/docs/ (accessed on 30 December 2021).

## Supplementary Materials

The following are available online at https://www.mdpi.com/article/10.3390/metabo12020122/s1, Figure S1: Upper Part: In the aldolase reaction, fructose-1,6-bisphosphate is converted to glyceraldehyde-3-phosphate and dihydroxyacetone phosphate. The 1st and 6th carbon atoms of fructose-1,6-bisphosphate are the phosphate carbons in their respective metabolites. Lower Part: In the second reaction an error is introduced that swaps the 1st and 3rd carbon atom of each metabolite, resulting in the 3rd and 4th carbon being the phosphate carbon, Table S1: Atom mapping model of the INCA E. coli model (Young, 2014), Table S2: Metabolic model of E. coli (see Table S1) with database identifiers, Table S3: Atom mapping model of E. coli (see Table S1) with MetAMDB atom mappings, Table S4: Flux estimation results for the E. coli model (see Table S1 (correct) and S7 (incorrect)), Table S5: Mass Isotopomer Distribution (MID) results for the E. coli model without errors (see Table S1), Table S6: Mass Isotopomer Distribution (MID) results for the E. coli model with MetAMDB atom mappings (see Table S3), Table S7: Atom mapping model of the INCA E. coli model (Young, 2014) with an error in R3, Table S8: Mass Isotopomer Distribution (MID) results for the E. coli model with an aldolase error (see Table S7).
