# Supplementary material for: MetAMDB: Metabolic Atom Mapping Database
Source: Metabolites. 2022 Jan 27;12(2):122. doi: 10.3390/metabo12020122 (PMC8878866; doi:10.3390/metabo12020122)
Supplement: Supplementary file 1 [file metabolites-12-00122-s001.zip › supplement_starke_wegner.pdf]

MetAMDB: Metabolic Atom Mapping Database

Collin Starke[1], *and Andre Wegner*[1*]

(1) Department of Bioinformatics and Biochemistry, Braunschweig Integrated Center of Systems Biology (BRICS), Technische Universität Braunschweig, Rebenring 56, 38106 Braunschweig, Germany

(*) To whom correspondence should be addressed (a.wegner@tu-bs.de)

This document contains the following information in support of the primary article:

Method Text: Flux estimation and MID simulation

All flux estimations and MID simulations were done in INCA (Young, 2014) for the provided *E. coli* model (Young et al., 2008). The model is a dynamic, nonsteady-state model and was used with its predetermined settings by INCA. For the flux set, fluxes were estimated and for the confidence interval calculation the INCA parameter continuation was used. Since the model is dynamic, multiple MIDs were simulated but only the last time point was used in this paper (0.0178).

Table S1: ***Atom mapping model of the INCA E. coli model (Young, 2014).***

ID	Substrates		Products
R1	G6P (abcdef)	<->	F6P (abcdef)
R2	F6P (abcdef)	->	FBP (abcdef)
R3	FBP (abcdef)	<->	DHAP (cba) + GAP (def)
R4	DHAP (abc)	<->	GAP (abc)
R5	GAP (abc)	<->	PG3 (abc)
R6	PG3 (abc)	<->	PEP (abc)
R7	PEP (abc)	->	Pyr (abc)
R8	G6P (abcdef)	->	PG6 (abcdef)
R9	PG6 (abcdef)	->	Ru5P (bcdef) + CO2 (a)
R10	Ru5P (abcde)	<->	X5P (abcde)
R11	Ru5P (abcde)	<->	R5P (abcde)
R12	X5P (abcde)	<->	GAP (cde) + EC2 (ab)
R13	F6P (abcdef)	<->	E4P (cdef) + EC2 (ab)
R14	S7P (abcdefg)	<->	R5P (cdefg) + EC2 (ab)
R15	F6P (abcdef)	<->	GAP (def) + EC3 (abc)
R16	S7P (abcdefg)	<->	E4P (defg) + EC3 (abc)
R17	PG6 (abcdef)	->	KDPG (abcdef)
R18	KDPG (abcdef)	->	Pyr (abc) + GAP (def)
R19	Pyr (abc)	->	AcCoA (bc) + CO2 (a)
R20	OAA (abcd) + AcCoA (ef)	->	Cit (dcbfea)
R21	Cit (abcdef)	<->	ICit (abcdef)
R22	ICit (abcdef)	<->	AKG (abcde) + CO2 (f)
R23	AKG (abcde)	->	SucCoA (bcde) + CO2 (a)
R24	SucCoA (abcd)	<->	Suc (abcd)
R25	Suc (abcd)	<->	Fum (abcd)
R26	Fum (abcd)	<->	Mal (abcd)
R27	Mal (abcd)	<->	OAA (abcd)
R28	Mal (abcd)	->	Pyr (abc) + CO2 (d)
R29	PEP (abc) + CO2 (d)	<->	OAA (abcd)
R30	AcCoA (ab)	<->	Ac (ab)
R31	DHAP (abc)	<->	Glyc3P (abc)
R32	Glyc3P (abc)	->	Glyc (abc)
R33	Glyc (abc)	->	HPA (abc)
R34	HPA (abc)	->	PDO (abc)
R35	AKG (abcde)	->	Glu (abcde)
R36	Glu (abcde)	->	Gln (abcde)
R37	Glu (abcde)	->	Pro (abcde)
R38	Glu (abcde) + CO2 (f) + Gln (ghijk) + Asp (lmno) + AcCoA (pq)	->	Arg (abcdef) + AKG (ghijk) + Fum (lmno) + Ac (pq)
R39	OAA (abcd) + Glu (efghi)	->	Asp (abcd) + AKG (efghi)
R40	Asp (abcd)	->	Asn (abcd)

R41	Pyr (abc) + Glu (defgh)	->	Ala (abc) + AKG (defgh)
R42	PG3 (abc) + Glu (defgh)	->	Ser (abc) + AKG (defgh)
R43	Ser (abc)	<->	Gly (ab) + MEETHF (c)
R44	Gly (ab)	<->	CO2 (a) + MEETHF (b)
R45	Thr (abcd)	->	Gly (ab) + AcCoA (cd)
R46	Ser (abc) + AcCoA (de)	->	Cys (abc) + Ac (de)
R47	Asp (abcd) + Pyr (efg) + Glu (hijkl) + SucCoA (mnop)	->	LL_DAP (abcdgfe) + AKG (hijkl) + Suc (mnop)
R48	LL_DAP (abcdefg)	->	Lys (abcdef) + CO2 (g)
R49	Asp (abcd)	->	Thr (abcd)
R50	Asp (abcd) + METHF (e) + Cys (fgh) + SucCoA (ijkl)	->	Met (abcde) + Pyr (fgh) + Suc (ijkl)
R51	Pyr (abc) + Pyr (def) + Glu (ghijk)	->	Val (abcef) + CO2 (d) + AKG (ghijk)
R52	AcCoA (ab) + Pyr (cde) + Pyr (fgh) + Glu (ijklm)	->	Leu (abdghe) + CO2 (c) + CO2 (f) + AKG (ijklm)
R53	Thr (abcd) + Pyr (efg) + Glu (hijkl)	->	Ile (abfcdg) + CO2 (e) + AKG (hijkl)
R54	PEP (abc) + PEP (def) + E4P (ghij) + Glu (klmno)	->	Phe (abcefghij) + CO2 (d) + AKG (klmno)
R55	PEP (abc) + PEP (def) + E4P (ghij) + Glu (klmno)	->	Tyr (abcefghij) + CO2 (d) + AKG (klmno)
R56	Ser (abc) + R5P (defgh) + PEP (ijk) + E4P (lmno) + PEP (pqr) + Gln (stuvw)	->	Trp (abcedklmnoj) + CO2 (i) + GAP (fgh) + Pyr (pqr) + Glu (stuvw)
R57	R5P (abcde) + FTHF (f) + Gln (ghijk) + Asp (lmno)	->	His (edcbaf) + AKG (ghijk) + Fum (lmno)
R58	MEETHF (a)	->	METHF (a)
R59	MEETHF (a)	->	FTHF (a)
R60	Gluc.pre (abcdef)	->	G6P (abcdef)
R61	Gluc.ext (abcdef)	->	G6P (abcdef)
R62	Cit.ext (abcdef)	->	Cit (abcdef)
R63	Glyc.ext (abc) + Dummy.ext	<->	Glyc (abc) + Dummy
R64	PDO (abc)	->	PDO.ext (abc)
R65	Ac (ab)	->	Ac.ext (ab)
R66	CO2 (a)	->	CO2.ext (a)
R67	0.488*Ala + 0.281*Arg + 0.229*Asn + 0.229*Asp + 0.087*Cys + 0.25*Glu + 0.25*Gln + 0.582*Gly + 0.09*His + 0.276*Ile + 0.428*Leu + 0.326*Lys + 0.146*Met + 0.176*Phe + 0.21*Pro + 0.205*Ser + 0.241*Thr + 0.054*Trp + 0.131*Tyr + 0.402*Val + 0.205*G6P + 0.071*F6P + 0.754*R5P + 0.129*GAP +	->	39.68*Biomass

	0.619*PG3 + 0.051*PEP + 0.083*Pyr + 2.51*AcCoA + 0.087*AKG + 0.34*OAA + 0.443*MEETHF		
R68	Dummy	->	Dummy.ext

Table S2: Metabolic model of E. coli (see Table S1) with database identifiers

reaction	substrates	arrow	products
v1 [PGLUCISOM-RXN]	G6P [Glucose-6-phosphate]	<->	F6P [CPD-18719]
v2 [6PFRUCTPHOS-RXN]	F6P [CPD-18719]	->	FBP [FRUCTOSE-16-DIPHOSPHATE]
v3 [F16ALDOLASE-RXN]	FBP [FRUCTOSE-16-DIPHOSPHATE]	<->	DHAP [136411] + GAP [GAP]
v4 [TRIOSEPISOMERIZATION-RXN]	DHAP [136411]	<->	GAP [GAP]
v5	GAP (abc) [GAP]	<->	PG3 (acb)
v6	PG3 (abc)	<->	PEP (cba) [PHOSPHO-ENOL-PYRUVATE]
v7 [PEPDEPHOS-RXN]	PEP [PHOSPHO-ENOL-PYRUVATE]	->	Pyr [PYRUVATE]
v8	G6P (abcdef)	->	PG6 (abcdef) [CPD-2961]
v9 [RXN-9952]	PG6 [CPD-2961]	->	Ru5P [RIBULOSE-5P] + CO2 [CARBON-DIOXIDE]
v10 [RIBULP3EPIM-RXN]	Ru5P [RIBULOSE-5P]	<->	X5P [XYLULOSE-5-PHOSPHATE]
v11 [RIB5PISOM-RXN]	Ru5P [RIBULOSE-5P]	<->	R5P [C00117]
v12	X5P (abcde) [XYLULOSE-5-PHOSPHATE]	↔	EC2 (ac) + GAP (ebd) [GAP]
v13	F6P (abcdef) [CPD-18719]	↔	EC2 (bf) + E4P (edca)
v14	S7P (abcdefg) [D-SEDOHEPTULOSE-7-P]	↔	EC2 (ab) + R5P (gfedc) [C00117]
v15	F6P (abcdef) [CPD-18719]	↔	EC3 (bfe) + GAP (dac) [GAP]
v16	S7P (abcdefg) [D-SEDOHEPTULOSE-7-P]	↔	EC3 (abc) + E4P (defg)
v17	PG6 [CPD-2961] (abcdef)	->	KDPG [2-DEHYDRO-3-DEOXY-D-GLUCONATE] (dacebf)
v18 [KDPGALDOL-RXN]	KDPG [2-KETO-3-DEOXY-6-P-GLUCONATE]	->	Pyr [PYRUVATE] + GAP [GAP]
v19 [PYRUVDEH-RXN]	Pyr [PYRUVATE]	->	AcCoA [ACETYL-COA] + CO2 [CARBON-DIOXIDE]
v20 [CITSYN-RXN]	OAA [OXALACETIC_ACID] + AcCoA [ACETYL-COA]	->	Cit [CIT]
v21	Cit [CIT] (abcdef)	<->	Icit [C00311] (afcbed)
v22 [ISOCITRATE-DEHYDROGENASE-NAD+-RXN]	Icit [C00311]	<->	AKG [2-KETOGLUTARATE] + CO2 [CARBON-DIOXIDE]
v23 [2OXOGLUTARATED	AKG [2-KETOGLUTARATE]	->	SucCoA [SUC-COA] + CO2 [CARBON-DIOXIDE]

EH-RXN]			
v24 [SUCCCOASYN-RXN]	SucCoA [SUC-COA]	<->	Suc [SUC]
v25 [SUCC-FUM-OXRED-RXN]	Suc [SUC]	<->	Fum [FUM]
v26 [FUMHYDR-RXN]	Fum [FUM]	<->	Mal [MAL]
v27 [MALATE-DEH-RXN]	Mal [MAL]	<->	OAA [OXALACETIC_ACID]
v28 [MALIC-NADP-RXN]	Mal [MAL]	->	Pyr [PYRUVATE] + CO2 [CARBON-DIOXIDE]
v29 [PEPCARBOX-RXN]	PEP [PHOSPHO-ENOL-PYRUVATE] + CO2 [HCO3]	<->	OAA [OXALACETIC_ACID]
v30 [ACETYL-COA-HYDROLASE-RXN]	AcCoA [ACETYL-COA]	<->	Ac [ACET]
v31 [BR22612]	DHAP [136411]	<->	Glyc3P [C00093]
v32 [RXN-14965]	Glyc3P [GLYCEROL-3P]	->	Glyc [GLYCEROL]
v33	Glyc (abc)	->	HPA (acb)
v34	HPA (abc)	->	PDO (abc)
v35	AKG (abcde) [2-KETOGLUTARATE]	->	Glu (ecabd)
v36	Glu (abcde)	->	Gln (abcde)
v37	Glu (abcde)	->	Pro (abcde)
v38	Glu (abcde) + CO2 (f) + Gln (ghijk) + Asp (lmno) + AcCoA (pq)	->	Arg (abcdef) + AKG (ijhkg) [2-KETOGLUTARATE] + Fum (mnlo) + Ac (pq)
v39	OAA (abcd) + Glu (efghi)	->	Asp (dbac) + AKG (ghfie) [2-KETOGLUTARATE]
v40	Asp (abcd)	->	Asn (abcd)
v41	Pyr (abc) + Glu (defgh)	->	Ala (cba) + AKG (fgehd) [2-KETOGLUTARATE]
v42	PG3 (abc) + Glu (defgh)	->	Ser (abc) + AKG (fgehd) [2-KETOGLUTARATE]
v43	Ser (abc)	<->	Gly (ab) + MEETHF (c)
v44	Gly (ab)	<->	CO2 (a) + MEETHF (b)
v45	Thr (abcd)	->	Gly (ab) + AcCoA (dc)
v46	Ser (abc) + AcCoA (de)	->	Cys (abc) + Ac (de)
v47	Asp (abcd) + Pyr (efg) + Glu (hijkl) + SucCoA (mnop)	->	LL_DAP (abcdefg) + AKG (jkilh) + Suc (mnop)
v48	LL_DAP (abcdefg)	->	Lys (abcdef) + CO2 (g)
v49	Asp (abcd)	->	Thr (abcd)
v50	Asp (abcd) + METHF (e) + Cys (fgh) + SucCoA (ijkl)	->	Met (abcde) + Pyr (hgf) + Suc (ijkl)
v51	Pyr (abc) + Pyr (def) + Glu (ghijk)	->	Val (cbaed) + CO2 (f) + AKG (ijhkg)
v52	AcCoA (ab) + Pyr (cde) + Pyr (fgh) + Glu (ijklm)	->	Leu (badgfc) + CO2 (e) + CO2 (h) + AKG (kljmi)

v53	Thr (abcd) + Pyr (efg) + Glu (hijkl)	->	Ile (abfcde) + CO2 (g) + AKG (jkilh)
v54	PEP (abc) + PEP (def) + E4P (ghij) + Glu (klmno)	->	Phe (cbaedghij) + CO2 (f) + AKG (mnlok)
v55	PEP (abc) + PEP (def) + E4P (ghij) + Glu (klmno)	->	Tyr (cbaedghij) + CO2 (f) + AKG (mnlok)
v56	Ser (abc) + R5P (defgh) + PEP (ijk) + E4P (lmno) + PEP (pqr) + Gln (stuvw)	->	Trp (abcghilmnoj) + CO2 (k) + GAP (fde) + Pyr (pqr) + Glu (stuvw)
v57	R5P (abcde) + FTHF (f) + Gln (ghijk) + Asp (lmno)	->	His (edcbaf) + AKG (ijhkg) + Fum (mnlo)
v58	MEETHF (a)	->	METHF (a)
v59	MEETHF (a)	->	FTHF (a)
v60	Gluc.pre (abcdef)	->	G6P (fedcba)
v61	Gluc.ext (abcdef)	->	G6P (fedcba)
v62	Cit.ext (abcdef)	->	Cit (abcdef)
v63	Glyc.ext (abc) + Dummy.ext	<->	Glyc (abc) + Dummy
v64	PDO (abc)	->	PDO.ext (abc)
v65	Ac (ab)	->	Ac.ext (ab)
v66	CO2 (a) [CARBON-DIOXIDE]	->	CO2.ext (a)
v67	0.488*Ala + 0.281*Arg + 0.229*Asn + 0.229*Asp + 0.087*Cys + 0.25*Glu + 0.25*Gln + 0.582*Gly + 0.09*His + 0.276*Ile + 0.428*Leu + 0.326*Lys + 0.146*Met + 0.176*Phe + 0.21*Pro + 0.205*Ser + 0.241*Thr + 0.054*Trp + 0.131*Tyr + 0.402*Val + 0.205*G6P + 0.071*F6P + 0.754*R5P + 0.129*GAP + 0.619*PG3 + 0.051*PEP + 0.083*Pyr + 2.51*AcCoA + 0.087*AKG + 0.34*OAA + 0.443*MEETHF	->	39.68*Biomass
v68	Dummy	->	Dummy.ext

Table S3: ***Atom mapping model of E. coli (see Table S1) with MetAMDB atom mappings.***

We utilized the *E. coli* atom mapping model in combination with MetAMDB reaction ids to generate MetAMDB atom mappings. Most model reactions are simplified, which means most atom mappings will stay similar to the original ones (with some reordered according to their InChI sorting). Some atom mappings were manually adjusted, which mainly include reduction of Acetyl-CoA and Succinyl-CoA. Simplified atom mappings only specify two and four carbon atoms respectively, while database atom mappings include all carbon atom in the mapping.

ID	Substrates		Products
R1	G6P (abcdef)	<->	F6P (afbcde)
R2	F6P (abcdef)	->	FBP (abcdef)
R3	FBP (abcdef)	<->	DHAP (ebf) + GAP (dac)
R4	DHAP (abc)	<->	GAP (abc)
R5	GAP (abc)	<->	PG3 (acb)
R6	PG3 (abc)	<->	PEP (cba)
R7	PEP (abc)	->	Pyr (abc)
R8	G6P (abcdef)	->	PG6 (abcdef)
R9	PG6 (abcdef)	->	Ru5P (eadbc) + CO2 (f)
R10	Ru5P (abcde)	<->	X5P (abcde)
R11	Ru5P (abcde)	<->	R5P (bdeca)
R12	X5P (abcde)	<->	EC2 (ac) + GAP (ebd)
R13	F6P (abcdef)	<->	EC2 (bf) + E4P (edca)
R14	S7P (abcdefg)	<->	EC2 (ab) + R5P (gfedc)
R15	F6P (abcdef)	<->	EC3 (bfe) + GAP (dac)
R16	S7P (abcdefg)	<->	EC3 (abc) + E4P (defg)
R17	PG6 (abcdef)	->	KDPG (dacebf)
R18	KDPG (abcdef)	->	Pyr (adf) + GAP (cbe)
R19	Pyr (abc)	->	AcCoA (ab) + CO2 (c)
R20	OAA (abcd) + AcCoA (tu)	->	Cit (eafcdb)
R21	Cit (abcdef)	<->	Icit (afcbed)
R22	ICit (abcdef)	<->	AKG (badcf) + CO2 (e)
R23	AKG (abcde)	->	SucCoA (badc) + CO2 (e)
R24	SucCoA (fghi)	<->	Suc (abcd)
R25	Suc (abcd)	<->	Fum (abcd)
R26	Fum (abcd)	<->	Mal (abcd)
R27	Mal (abcd)	<->	OAA (abcd)
R28	Mal (abcd)	->	Pyr (abd) + CO2 (c)
R29	PEP (abc) + CO2 (d)	<->	OAA (abdc)
R30	AcCoA (ab)	<->	Ac (ab)
R31	DHAP (abc)	<->	Glyc3P (abc)
R32	Glyc3P (abc)	->	Glyc (abc)
R33	Glyc (abc)	->	HPA (acb)
R34	HPA (abc)	->	PDO (abc)
R35	AKG (abcde)	->	Glu (ecabd)
R36	Glu (abcde)	->	Gln (abcde)

R37	Glu (abcde)	->	Pro (abcde)
R38	Glu (abcde) + CO2 (f) + Gln (ghijk) + Asp (lmno) + AcCoA (pq)	->	Arg (abcdef) + AKG (ijhkg) + Fum (mnlo) + Ac (pq)
R39	OAA (abcd) + Glu (efghi)	->	Asp (dbac) + AKG (ghfie)
R40	Asp (abcd)	->	Asn (abcd)
R41	Pyr (abc) + Glu (defgh)	->	Ala (cba) + AKG (fgehd)
R42	PG3 (abc) + Glu (defgh)	->	Ser (abc) + AKG (fgehd)
R43	Ser (abc)	<->	Gly (ab) + MEETHF (c)
R44	Gly (ab)	<->	CO2 (a) + MEETHF (b)
R45	Thr (abcd)	->	Gly (ab) + AcCoA (dc)
R46	Ser (abc) + AcCoA (de)	->	Cys (abc) + Ac (de)
R47	Asp (abcd) + Pyr (efg) + Glu (hijkl) + SucCoA (mnop)	->	LL_DAP (abcdefg) + AKG (jkilh) + Suc (mnop)
R48	LL_DAP (abcdefg)	->	Lys (abcdef) + CO2 (g)
R49	Asp (abcd)	->	Thr (abcd)
R50	Asp (abcd) + METHF (e) + Cys (fgh) + SucCoA (ijkl)	->	Met (abcde) + Pyr (hgf) + Suc (ijkl)
R51	Pyr (abc) + Pyr (def) + Glu (ghijk)	->	Val (cbaed) + CO2 (f) + AKG (ijhkg)
R52	AcCoA (ab) + Pyr (cde) + Pyr (fgh) + Glu (ijklm)	->	Leu (badgfc) + CO2 (e) + CO2 (h) + AKG (kljmi)
R53	Thr (abcd) + Pyr (efg) + Glu (hijkl)	->	Ile (abfcde) + CO2 (g) + AKG (jkilh)
R54	PEP (abc) + PEP (def) + E4P (ghij) + Glu (klmno)	->	Phe (cbaedghij) + CO2 (f) + AKG (mnlok)
R55	PEP (abc) + PEP (def) + E4P (ghij) + Glu (klmno)	->	Tyr (cbaedghij) + CO2 (f) + AKG (mnlok)
R56	Ser (abc) + R5P (defgh) + PEP (ijk) + E4P (lmno) + PEP (pqr) + Gln (stuvw)	->	Trp (abcghilmnoj) + CO2 (k) + GAP (fde) + Pyr (pqr) + Glu (stuvw)
R57	R5P (abcde) + FTHF (f) + Gln (ghijk) + Asp (lmno)	->	His (edcbaf) + AKG (ijhkg) + Fum (mnlo)
R58	MEETHF (a)	->	METHF (a)
R59	MEETHF (a)	->	FTHF (a)
R60	Gluc.pre (abcdef)	->	G6P (fedcba)
R61	Gluc.ext (abcdef)	->	G6P (fedcba)
R62	Cit.ext (abcdef)	->	Cit (abcdef)
R63	Glyc.ext (abc) + Dummy.ext	<->	Glyc (abc)
R64	PDO (abc)	->	PDO.ext (abc)
R65	Ac (ab)	->	Ac.ext (ab)
R66	CO2 (a)	->	CO2.ext (a)

	0.488*Ala + 0.281*Arg +		
	0.229*Asn + 0.229*Asp +		
	0.087*Cys + 0.25*Glu + 0.25*Gln		
	+ 0.582*Gly + 0.09*His +		
	0.276*Ile + 0.428*Leu +		
	0.326*Lys + 0.146*Met +		
	0.176*Phe + 0.21*Pro + 0.205*Ser		
	+ 0.241*Thr + 0.054*Trp +		
	0.131*Tyr + 0.402*Val +		
	0.205*G6P + 0.071*F6P +		
	0.754*R5P + 0.129*GAP +		
	0.619*PG3 + 0.051*PEP +		
	0.083*Pyr + 2.51*AcCoA +		
	0.087*AKG + 0.34*OAA +		
R67	0.443*MEETHF	->	39.68*Biomass
R68	Dummy	->	Dummy.ext

Table S4: ***Flux estimation results for the E. coli model (see Table S1 (correct) and S7 (incorrect)).***

Reaction				Cor.	Model		Incor.	Model
			Flux	95%	Conf.	Flux	95%	Conf.
G6P ↔ F6P	R1	net	63.91	52.42	72.28	77.10	NaN	86.21
		exch	0.00	0.00	99.54	291.10	242.16	338.20
F6P → FBP	R2	net	86.24	79.23	92.91	76.40	NaN	86.12
FBP ↔ DHAP + GAP	R3	net	86.24	79.23	92.91	76.40	NaN	86.12
		exch	595.57	0.00	Inf	271.00	0.00	4.20E+6
DHAP ↔ GAP	R4	net	-41.07	-48.47	-33.94	-42.00	-52.04	-31.60
		exch	246.39	189.76	349.02	31.90	28.17	35.88
GAP ↔ PG3	R5	net	56.28	49.92	64.40	57.70	52.00	64.56
		exch	8.03E+5	165.61	Inf	4.14E+5	0.00	Inf
PG3 ↔ PEP	R6	net	54.72	49.11	60.24	56.40	50.56	63.19
		exch	5571.90	153.76	Inf	5.23E+5	0.00	Inf
PEP → Pyr	R7		48.58	43.22	56.55	53.50	48.05	60.20
G6P → PG6	R8		34.69	28.87	45.02	23.70	21.09	26.52
PG6 → Ru5P + CO2	R9		34.55	28.65	45.54	0.00	0.00	1.52
Ru5P ↔ X5P	R10	net	22.39	18.45	30.70	-0.60	-0.64	0.55
		exch	108.87	0.00	Inf	4.73E+6	9.35E+4	NaN
Ru5P ↔ R5P	R11	net	12.16	10.25	16.50	0.60	0.00	1.04
		exch	0.00	0.00	Inf	4.73E+6	6.04E+4	NaN
X5P ↔ EC2 + GAP	R12	net	22.39	18.45	30.70	-0.60	-0.64	0.55
		exch	3125.70	47.20	Inf	4.73E+6	6.17E+4	NaN
F6P ↔ EC2 + E4P	R13	net	-11.03	-15.19	-9.12	0.40	-0.21	0.48
		exch	5.80	0.00	13.28	61.30	49.75	76.10
S7P ↔ EC2 + R5P	R14	net	-11.36	-15.72	-9.35	0.10	-0.35	0.16
		exch	127.32	0.00	Inf	2.95E+6	1.75E+4	Inf
F6P ↔ EC3 + GAP	R15	net	-11.36	-15.72	-9.35	0.10	-0.35	0.16
		exch	0.00	0.00	Inf	2.21E+6	7316.10	Inf
S7P ↔ EC3 + E4P	R16	net	11.36	9.35	15.72	-0.10	-0.16	0.35
		exch	0.00	0.00	Inf	2.95E+6	1.71E+4	2.95E+6
PG6 → KDPG	R17		0.14	0.00	0.88	23.70	NaN	26.71
KDPG → Pyr + GAP	R18		0.14	0.00	0.88	23.70	NaN	26.71
Pyr → AcCoA + CO2	R19		49.52	44.22	57.68	75.20	NaN	84.43

OAA + AcCoA → Cit	R20		46.58	41.12	51.84	72.70	65.44	81.61
Cit ↔ Icit	R21	net	46.85	41.45	54.49	72.90	65.32	NaN
		exch	94.63	45.78	238.21	1.29E+4	172.23	NaN
Icit ↔ AKG + CO2	R22	net	46.85	41.45	54.49	72.90	65.32	NaN
		exch	1.85E+4	47.91	Inf	9137.60	184.37	Inf
AKG → SucCoA + CO2	R23		45.89	40.52	53.87	72.10	65.35	81.24
SucCoA ↔ Suc	R24	net	45.46	40.02	53.42	71.70	64.77	80.53
		exch	451.66	0.00	Inf	85.70	0.00	Inf
Suc ↔ Fum	R25	net	45.89	40.52	53.87	72.10	65.35	81.24
		exch	146.97	53.13	7453.40	4.34E+5	6.83	4.34E+5
Fum ↔ Mal	R26	net	46.22	40.80	54.14	72.30	64.64	81.63
		exch	5.07E+5	196.33	Inf	8.67E+5	610.91	Inf
Mal ↔ OAA	R27	net	43.07	37.66	48.28	72.30	64.68	81.57
		exch	198.16	134.64	2.81E+4	9.31E+5	477.61	Inf
Mal → Pyr + CO2	R28		3.15	1.85	4.02	0.00	0.00	0.33
PEP + CO2 ↔ OAA	R29	net	5.44	4.06	6.43	2.20	1.72	2.57
		exch	7.14	5.31	9.06	0.00	0.00	0.75
AcCOA ↔ Ac	R30	net	-0.15	-0.21	-0.05	-0.10	-0.16	0.01
		exch	0.00	0.00	Inf	3.65E+5	0.00	3.65E+5
DHAP ↔ Glyc3P	R31	net	127.32	114.03	140.16	118.50	100.25	137.60
		exch	3.00E+5	0.00	Inf	4.30	0.00	Inf
Glyc3P → Glyc	R32		127.32	114.03	140.16	118.50	100.25	137.60
Glyc → HPA	R33		129.30	116.61	141.99	120.50	NaN	139.48
HPA → PDO	R34		129.30	116.61	141.99	120.50	NaN	139.48
AKG → Glu	R35		5.84	4.95	6.49	5.20	NaN	5.96
Glu → Gln	R36		0.60	0.51	0.67	0.50	0.43	0.60
Glu → Pro	R37		0.19	0.16	0.21	0.20	0.13	0.19
Glu + CO2 + Gln + Asp + AcCoA → Arg + AKG + Fum + Ac	R38		0.25	0.21	0.28	0.20	0.18	0.25
OAA + Glu → Asp + AKG	R39		1.63	1.38	1.81	1.60	NaN	1.76
Asp → Asn	R40		0.20	0.17	0.23	0.20	0.14	0.20
Pyr + Glu → Ala + AKG	R41		0.44	0.37	0.48	0.40	0.31	0.44
PG3 + Glu → Ser + AKG	R42		1.00	0.84	1.12	0.80	0.63	0.94
Ser ↔ Gly + MEETHF	R43	net	0.56	0.47	0.63	0.40	NaN	0.49
		exch	0.84	0.73	0.97	1.70	1.29	1.97

Gly ↔ CO_2 + MEETHF	R44	net	0.04	0.04	0.07	0.10	0.07	0.15
		exch	0.17	0.13	0.20	0.00	0.00	0.07
Thr → Gly + AcCoA	R45		0.00	0.00	0.06	0.20	NaN	0.21
Ser + AcCoA → Cys + Ac	R46		0.21	0.18	0.23	0.20	0.15	0.21
Asp + Pyr + Glu + SucCoA → LL_DAP + AKG + Suc	R47		0.29	0.25	0.32	0.30	0.21	0.29
LL_DAP → Lys + CO_2	R48		0.29	0.25	0.32	0.30	0.21	0.29
Asp → Thr	R49		0.46	0.39	0.54	0.60	0.42	0.66
Asp + METHF + Cys + SucCoA → Met + Pyr + Suc	R50		0.13	0.11	0.14	0.10	0.09	0.13
Pyr + Pyr + Glu → Val + CO_2 + AKG	R51		0.36	0.30	0.40	0.30	0.25	0.36
AcCoA + Pyr + Pyr + Glu → Leu + CO_2 + CO_2 + AKG	R52		0.38	0.32	0.42	0.30	0.27	0.38
Thr + Pyr + Glu → Ile + CO_2 + AKG	R53		0.25	0.21	0.27	0.20	0.17	0.25
PEP + PEP + E4P + Glu → Phe + CO_2 + AKG	R54		0.16	0.13	0.17	0.10	0.11	0.16
PEP + PEP + E4P + Glu → Tyr + CO_2 + AKG	R55		0.12	0.10	0.13	0.10	0.08	0.12
Ser + R5P + PEP + E4P + PEP + Gln → Trp + CO_2 + GAP + Pyr + Glu	R56		0.05	0.04	0.05	0.00	0.03	0.05
R5P + FTHF + Gln + Asp → His + AKG + Fum	R57		0.08	0.07	0.09	0.10	0.06	0.08
MEETHF → METHF	R58		0.13	0.11	0.14	0.10	0.09	0.13
MEETHF → FTHF	R59		0.08	0.07	0.09	0.10	0.06	0.08
Gluc.pre → G6P	R60		6.44	5.39	7.37	0.00	0.00	0.04
Gluc.ext → G6P	R61		92.35	86.05	98.54	101.00	91.30	110.98
Cit.ext → Cit	R62		0.27	0.24	0.30	0.20	NaN	0.23
Glyc.ext ↔ Glyc	R63	net	1.98	1.76	2.20	1.99	1.65	2.27
		exch	45.83	0.00	Inf	1500.00	0.00	Inf
PDO → PDO.ext	R64		129.30	116.61	141.99	120.50	NaN	139.48
Ac → Ac.ext	R65		0.31	0.28	0.35	0.30	0.26	0.36
CO_2 → CO_2.ext	R66		176.29	158.33	205.05	219.60	197.85	NaN
Biomass formation	R67		0.89	0.76	0.99	0.80	0.63	0.89
Dummy → Dummy.ext	R68		1.98	1.76	2.22	2.00	1.65	2.27

Table S5: ***Mass Isotopomer Distribution (MID) results for the E. coli model without errors (see Table S1).***

Mass Isotopomer	M0	M1	M2	M3	M4	M5	M6	M7	M8	M9
AKG5	0.1781	0.2673	0.2766	0.1839	0.0728	0.0213				
AcCoA	0.5497	0.2209	0.2294							
Ala2	0.5617	0.2153	0.223							
Ala3	0.5295	0.2285	0.0452	0.1967						
Asp2a	0.4995	0.2993	0.2012							
Asp2b	0.4995	0.2993	0.2012							
Asp3	0.3156	0.3617	0.2292	0.0935						
Asp4	0.2447	0.3257	0.2443	0.1349	0.0504					
Cit6	0.1157	0.2308	0.2688	0.2156	0.117	0.0424	0.0097			
E4P	0.5423	0.1861	0.0469	0.0956	0.1291					
Glu4	0.2276	0.3181	0.2802	0.1341	0.0401					
Glu5	0.1794	0.267	0.2762	0.1834	0.0726	0.0213				
Gly1	0.726	0.274								
Gly2	0.6531	0.1417	0.2052							
Ile5a	0.1737	0.2686	0.2782	0.1849	0.0732	0.0214				
Ile5b	0.1737	0.2686	0.2782	0.1849	0.0732	0.0214				
Leu5	0.1787	0.2669	0.2749	0.1847	0.0728	0.0221				
Mal4	0.2412	0.3244	0.2511	0.1349	0.0485					
Met4a	0.1916	0.3436	0.2812	0.1468	0.0368					
Met4b	0.1916	0.3436	0.2812	0.1468	0.0368					
Met5	0.1486	0.2939	0.2763	0.1779	0.0836	0.0198				
Phe2	0.7178	0.068	0.2142							
Phe8a	0.1696	0.1841	0.2227	0.1498	0.1243	0.0737	0.051	0.0177	0.007	
Phe8b	0.1696	0.1841	0.2227	0.1498	0.1243	0.0737	0.051	0.0177	0.007	
Phe9	0.1612	0.1832	0.1608	0.1706	0.1307	0.093	0.0529	0.03	0.0113	0.0063
Pyr3	0.5169	0.2342	0.0467	0.2023						
Ser2a	0.5055	0.3183	0.1762							
Ser2b	0.6885	0.1014	0.2101							
Ser2c	0.5055	0.3183	0.1762							
Suc4	0.2376	0.323	0.2581	0.1348	0.0465					
Thr3	0.3157	0.3616	0.2291	0.0935						
Thr4	0.2448	0.3257	0.2442	0.1349	0.0504					
Tyr2	0.7179	0.068	0.2141							
Val4	0.3024	0.2428	0.3009	0.1013	0.0526					
Val5	0.2844	0.2429	0.1959	0.1752	0.0554	0.0464				

Table S6: ***Mass Isotopomer Distribution (MID) results for the E. coli model with MetAMDB atom mappings (see Table S3).***

Mass Isotopomer	M0	M1	M2	M3	M4	M5	M6	M7	M8	M9
AKG5	0.1781	0.2673	0.2766	0.1839	0.0728	0.0213				
AcCoA	0.5497	0.2209	0.2294							
Ala2	0.5617	0.2153	0.223							
Ala3	0.5295	0.2285	0.0452	0.1967						
Asp2a	0.4995	0.2993	0.2012							
Asp2b	0.4995	0.2993	0.2012							
Asp3	0.3156	0.3617	0.2292	0.0935						
Asp4	0.2447	0.3257	0.2443	0.1349	0.0504					
Cit6	0.1157	0.2308	0.2688	0.2156	0.117	0.0424	0.0097			
E4P	0.5423	0.1861	0.0469	0.0956	0.1291					
Glu4	0.2276	0.3181	0.2802	0.1341	0.0401					
Glu5	0.1794	0.267	0.2762	0.1834	0.0726	0.0213				
Gly1	0.726	0.274								
Gly2	0.6531	0.1417	0.2052							
Ile5a	0.1737	0.2686	0.2782	0.1849	0.0732	0.0214				
Ile5b	0.1737	0.2686	0.2782	0.1849	0.0732	0.0214				
Leu5	0.1787	0.2669	0.2749	0.1847	0.0728	0.0221				
Mal4	0.2412	0.3244	0.2511	0.1349	0.0485					
Met4a	0.1916	0.3436	0.2812	0.1468	0.0368					
Met4b	0.1916	0.3436	0.2812	0.1468	0.0368					
Met5	0.1486	0.2939	0.2763	0.1779	0.0836	0.0198				
Phe2	0.7178	0.068	0.2142							
Phe8a	0.1696	0.1841	0.2227	0.1498	0.1243	0.0737	0.051	0.0177	0.007	
Phe8b	0.1696	0.1841	0.2227	0.1498	0.1243	0.0737	0.051	0.0177	0.007	
Phe9	0.1612	0.1832	0.1608	0.1706	0.1307	0.093	0.0529	0.03	0.0113	0.0063
Pyr3	0.5169	0.2342	0.0467	0.2023						
Ser2a	0.5055	0.3183	0.1762							
Ser2b	0.6885	0.1014	0.2101							
Ser2c	0.5055	0.3183	0.1762							
Suc4	0.2376	0.323	0.2581	0.1348	0.0465					
Thr3	0.3157	0.3616	0.2291	0.0935						
Thr4	0.2448	0.3257	0.2442	0.1349	0.0504					
Tyr2	0.7179	0.068	0.2141							
Val4	0.3024	0.2428	0.3009	0.1013	0.0526					
Val5	0.2844	0.2429	0.1959	0.1752	0.0554	0.0464				

Table S7: ***Atom mapping model of the INCA E. coli model (Young, 2014) with an error in R3.***

In this model we introduced an error in the aldolase reaction with the ID R3. Both dihydroxyacetone phosphate's (DHAP) and glyceraldehyde 3-phosphate's (GAP) mappings are mirrored, meaning the first and third carbon atoms are swapped in each metabolite.

ID	Substrates		Products
R1	G6P (abcdef)	<->	F6P (abcdef)
R2	F6P (abcdef)	->	FBP (abcdef)
**R3**	**FBP (abcdef)**	**<->**	**DHAP (abc) + GAP (fed)**
R4	DHAP (abc)	<->	GAP (abc)
R5	GAP (abc)	<->	PG3 (abc)
R6	PG3 (abc)	<->	PEP (abc)
R7	PEP (abc)	->	Pyr (abc)
R8	G6P (abcdef)	->	PG6 (abcdef)
R9	PG6 (abcdef)	->	Ru5P (bcdef) + CO2 (a)
R10	Ru5P (abcde)	<->	X5P (abcde)
R11	Ru5P (abcde)	<->	R5P (abcde)
R12	X5P (abcde)	<->	GAP (cde) + EC2 (ab)
R13	F6P (abcdef)	<->	E4P (cdef) + EC2 (ab)
R14	S7P (abcdefg)	<->	R5P (cdefg) + EC2 (ab)
R15	F6P (abcdef)	<->	GAP (def) + EC3 (abc)
R16	S7P (abcdefg)	<->	E4P (defg) + EC3 (abc)
R17	PG6 (abcdef)	->	KDPG (abcdef)
R18	KDPG (abcdef)	->	Pyr (abc) + GAP (def)
R19	Pyr (abc)	->	AcCoA (bc) + CO2 (a)
R20	OAA (abcd) + AcCoA (ef)	->	Cit (dcbfea)
R21	Cit (abcdef)	<->	ICit (abcdef)
R22	ICit (abcdef)	<->	AKG (abcde) + CO2 (f)
R23	AKG (abcde)	->	SucCoA (bcde) + CO2 (a)
R24	SucCoA (abcd)	<->	Suc (abcd)
R25	Suc (abcd)	<->	Fum (abcd)
R26	Fum (abcd)	<->	Mal (abcd)
R27	Mal (abcd)	<->	OAA (abcd)
R28	Mal (abcd)	->	Pyr (abc) + CO2 (d)
R29	PEP (abc) + CO2 (d)	<->	OAA (abcd)
R30	AcCoA (ab)	<->	Ac (ab)
R31	DHAP (abc)	<->	Glyc3P (abc)
R32	Glyc3P (abc)	->	Glyc (abc)
R33	Glyc (abc)	->	HPA (abc)
R34	HPA (abc)	->	PDO (abc)
R35	AKG (abcde)	->	Glu (abcde)
R36	Glu (abcde)	->	Gln (abcde)
R37	Glu (abcde)	->	Pro (abcde)
R38	Glu (abcde) + CO2 (f) + Gln (ghijk) + Asp (lmno) + AcCoA	->	Arg (abcdef) + AKG (ghijk) + Fum (lmno) + Ac (pq)

	(pq)		
R39	OAA (abcd) + Glu (efghi)	->	Asp (abcd) + AKG (efghi)
R40	Asp (abcd)	->	Asn (abcd)
R41	Pyr (abc) + Glu (defgh)	->	Ala (abc) + AKG (defgh)
R42	PG3 (abc) + Glu (defgh)	->	Ser (abc) + AKG (defgh)
R43	Ser (abc)	<->	Gly (ab) + MEETHF (c)
R44	Gly (ab)	<->	CO2 (a) + MEETHF (b)
R45	Thr (abcd)	->	Gly (ab) + AcCoA (cd)
R46	Ser (abc) + AcCoA (de)	->	Cys (abc) + Ac (de)
R47	Asp (abcd) + Pyr (efg) + Glu (hijkl) + SucCoA (mnop)	->	LL_DAP (abcdgfe) + AKG (hijkl) + Suc (mnop)
R48	LL_DAP (abcdefg)	->	Lys (abcdef) + CO2 (g)
R49	Asp (abcd)	->	Thr (abcd)
R50	Asp (abcd) + METHF (e) + Cys (fgh) + SucCoA (ijkl)	->	Met (abcde) + Pyr (fgh) + Suc (ijkl)
R51	Pyr (abc) + Pyr (def) + Glu (ghijk)	->	Val (abcef) + CO2 (d) + AKG (ghijk)
R52	AcCoA (ab) + Pyr (cde) + Pyr (fgh) + Glu (ijklm)	->	Leu (abdghe) + CO2 (c) + CO2 (f) + AKG (ijklm)
R53	Thr (abcd) + Pyr (efg) + Glu (hijkl)	->	Ile (abfcdg) + CO2 (e) + AKG (hijkl)
R54	PEP (abc) + PEP (def) + E4P (ghij) + Glu (klmno)	->	Phe (abcefghij) + CO2 (d) + AKG (klmno)
R55	PEP (abc) + PEP (def) + E4P (ghij) + Glu (klmno)	->	Tyr (abcefghij) + CO2 (d) + AKG (klmno)
R56	Ser (abc) + R5P (defgh) + PEP (ijk) + E4P (lmno) + PEP (pqr) + Gln (stuvw)	->	Trp (abcedklmnoj) + CO2 (i) + GAP (fgh) + Pyr (pqr) + Glu (stuvw)
R57	R5P (abcde) + FTHF (f) + Gln (ghijk) + Asp (lmno)	->	His (edcbaf) + AKG (ghijk) + Fum (lmno)
R58	MEETHF (a)	->	METHF (a)
R59	MEETHF (a)	->	FTHF (a)
R60	Gluc.pre (abcdef)	->	G6P (abcdef)
R61	Gluc.ext (abcdef)	->	G6P (abcdef)
R62	Cit.ext (abcdef)	->	Cit (abcdef)
R63	Glyc.ext (abc) + Dummy.ext	<->	Glyc (abc) + Dummy
R64	PDO (abc)	->	PDO.ext (abc)
R65	Ac (ab)	->	Ac.ext (ab)
R66	CO2 (a)	->	CO2.ext (a)
R67	0.488*Ala + 0.281*Arg + 0.229*Asn + 0.229*Asp + 0.087*Cys + 0.25*Glu + 0.25*Gln + 0.582*Gly + 0.09*His + 0.276*Ile + 0.428*Leu + 0.326*Lys + 0.146*Met + 0.176*Phe + 0.21*Pro + 0.205*Ser + 0.241*Thr + 0.054*Trp +	->	39.68*Biomass

	0.131*Tyr + 0.402*Val + 0.205*G6P + 0.071*F6P + 0.754*R5P + 0.129*GAP + 0.619*PG3 + 0.051*PEP + 0.083*Pyr + 2.51*AcCoA + 0.087*AKG + 0.34*OAA + 0.443*MEETHF		
R68	Dummy	->	Dummy.ext

Table S8: ***Mass Isotopomer Distribution (MID) results for the E. coli model with an aldolase error (see Table S7).***

Mass Isotopomer	M0	M1	M2	M3	M4	M5	M6	M7	M8	M9
AKG5	0.1957	0.2761	0.2773	0.1687	0.0661	0.0162				
AcCoA	0.5826	0.1785	0.2388							
Ala2	0.6084	0.1681	0.2236							
Ala3	0.5027	0.1837	0.1182	0.1953						
Asp2a	0.5064	0.3101	0.1836							
Asp2b	0.5064	0.3101	0.1836							
Asp3	0.3327	0.3728	0.2264	0.068						
Asp4	0.254	0.333	0.25	0.1305	0.0324					
Cit6	0.1264	0.2473	0.2767	0.2072	0.1026	0.034	0.0058			
E4P	0.3974	0.2889	0.0608	0.1644	0.0884					
Glu4	0.2496	0.3347	0.2557	0.1292	0.0308					
Glu5	0.1963	0.2759	0.2771	0.1685	0.066	0.0162				
Gly1	0.71	0.29								
Gly2	0.636	0.1319	0.2321							
Ile5a	0.1939	0.2766	0.2779	0.1691	0.0662	0.0163				
Ile5b	0.1939	0.2766	0.2779	0.1691	0.0662	0.0163				
Leu5	0.2194	0.2546	0.273	0.1655	0.067	0.0204				
Mal4	0.254	0.3331	0.25	0.1305	0.0325					
Met4a	0.2331	0.3608	0.2703	0.1154	0.0204					
Met4b	0.2331	0.3608	0.2703	0.1154	0.0204					
Met5	0.178	0.3094	0.2748	0.1663	0.0618	0.0097				
Phe2	0.6693	0.0853	0.2454							
Phe8a	0.178	0.1748	0.1937	0.1942	0.1148	0.0853	0.0402	0.0136	0.0053	
Phe8b	0.178	0.1748	0.1937	0.1942	0.1148	0.0853	0.0402	0.0136	0.0053	
Phe9	0.1706	0.165	0.1352	0.2052	0.1316	0.085	0.0654	0.0246	0.0123	0.005
Pyr3	0.4709	0.1937	0.1267	0.2087						
Ser2a	0.5536	0.3078	0.1386							
Ser2b	0.6469	0.1166	0.2365							
Ser2c	0.5536	0.3078	0.1386							
Suc4	0.254	0.3331	0.25	0.1305	0.0325					
Thr3	0.3327	0.3728	0.2264	0.068						
Thr4	0.254	0.333	0.25	0.1305	0.0324					
Tyr2	0.6723	0.0847	0.243							
Val4	0.3398	0.2077	0.3102	0.0852	0.0571					
Val5	0.2745	0.1968	0.2209	0.1905	0.0675	0.0499				

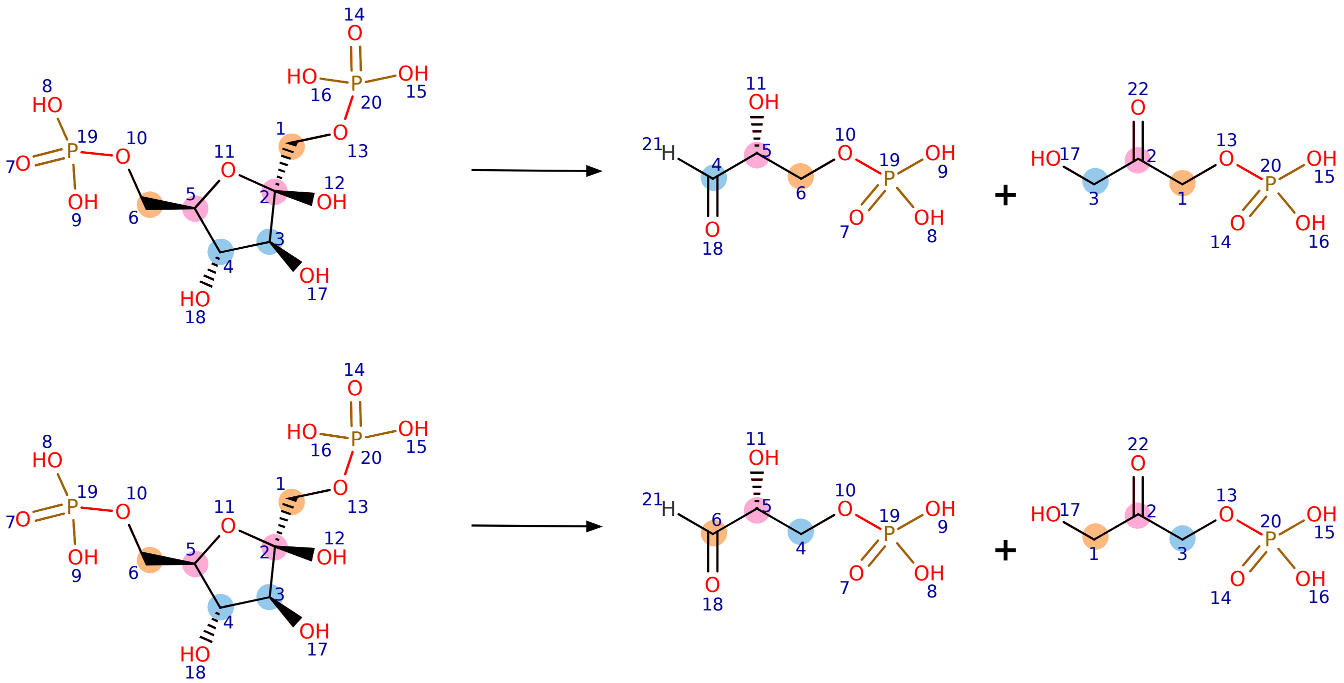

Figure S1: Upper Part: In the aldolase reaction, fructose-1,6-bisphosphate is converted to glyceraldehyde-3-phosphate and dihydroxyacetone phosphate. The 1st and 6th carbon atoms of fructose-1,6-bisphosphate are the phosphate carbons in their respective metabolites. Lower Part: In the second reaction an error is introduced that swaps the 1st and 3rd carbon atom of each metabolite, resulting in the 3rd and 4th carbon being the phosphate carbon.
